# The Work‐Averse Cyberattacker Model: Theory and Evidence from Two Million Attack Signatures

**DOI:** 10.1111/risa.13732

**Published:** 2021-05-07

**Authors:** Luca Allodi, Fabio Massacci, Julian Williams

**Affiliations:** ^1^ Technical University of Eindhoven Groene Loper 5 Eindhoven The Netherlands; ^2^ University of Trento Via Sommarive 9 Povo (Trento) Italy; ^3^ Vrije Universiteit Amsterdam De Boelelaan 1111 Amsterdam The Netherlands; ^4^ Durham University Business School Mill Hill Lane Durham UK

**Keywords:** Cyber security, hackers model, risk management, update costs

## Abstract

The assumption that a cyberattacker will potentially exploit all present vulnerabilities drives most modern cyber risk management practices and the corresponding security investments. We propose a new attacker model, based on dynamic optimization, where we demonstrate that large, initial, fixed costs of exploit development induce attackers to delay implementation and deployment of exploits of vulnerabilities. The theoretical model predicts that mass attackers will preferably (i) exploit only one vulnerability per software version, (ii) largely include only vulnerabilities requiring low attack complexity, and (iii) be slow at trying to weaponize new vulnerabilities . These predictions are empirically validated on a large data set of observed massed attacks launched against a large collection of information systems. Findings in this article allow cyber risk managers to better concentrate their efforts for vulnerability management, and set a new theoretical and empirical basis for further research defining attacker (offensive) processes.

## Graphical abstract

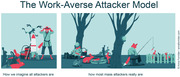

## 1 INTRODUCTION

A natural starting point for an evidence, big‐data based cyber‐risk model is to look at “attacks in the wild”: Each attempt to attack a system using a vulnerability and an exploit mechanism generates a specific *attack signature*, which may be recorded by software security vendors and can be identified by security researchers (Bilge & Dumitras, 2012) and linked to vulnerabilities that attackers seek to exploit (Allodi & Massacci, 2014).

For example, attackers focusing on chip and pin credit cards, which require physical access, are proactive and rapidly update their small menu of exploits (Murdoch, Drimer, Anderson, & Bond, 2010). In contrast, attackers on the web seem to be wary of exploiting the full range of vulnerabilities available to them: The actual risk of attacks in the wild is limited to 100 vulnerabilities out of the 50,000 reported in vulnerability databases (Allodi & Massacci, 2014; Nayak, Marino, Efstathopoulos, & Dumitraş, 2014). Even untimely disclosures do not seem to increase attack volumes (Mitra & Ransbotham, 2015).

This empirical evidence of web attacker behavior is at odds with the attacker models that underpin most cyber‐risk models: A system should be secured “against arbitrary behavior of the saboteur” (Dolev & Yao, 1983). Variants of the all‐powerful attacker model exist (e.g., honest‐but‐curious, game‐based models) but they only changed the power and speed of attacks not the will: If there is a vulnerability that the attacker can exploit, she will eventually do it.

As a result, current cyber‐risk standards (e.g., US NIST‐800, UK IAS) provide advice based on vulnerability severity: All severe vulnerabilities present in a system should be addressed ((e.g., the comments by Schneier, 2008, that cover similar ground). Indeed, papers on web security report the persistence of vulnerabilities on internet sites as evidence for risk underestimation by website owners (Nikiforakis et al., 2014; Stock, Lekies, & Johns, 2013). While sound in the presence of limited information, this advice often yields disproportionate mitigations, which only address threat inflation by security vendors (Brito & Watkins, 2011). Big data on attacks may allow us to use more accurate models of attackers and empirically validate them. Such models will then provide a better cyber‐risk assessment strategy for defenders.

The key contribution of this article is a novel theoretical model of the dynamic decisions of the attacker based on Stokey's logic of inaction (Stokey, 2008). We call attackers “*work averse*” to capture the natural assumption that attackers will not engineer and adopt new, complex exploits if they can obtain a satisfactory result with what they already have. If this assumption empirically holds, attackers will flock to the set of low‐complexity vulnerabilities with high impact, and postpone the adoption of new exploits until the previous ones become ineffective (e.g., as most vulnerable systems get patched). The proposed model steers away from classical, and empirically disproved (e.g., Allodi, 2015; Nayak et al., 2014) assumptions on the production function of new cyberattacks, and reconciles these empirical observations with a novel model describing the arrival process of new attacks at scale. Our model has profound implications for practical cyber‐risk management; for example, our model indicates that once an attack for a vulnerability in a software is deployed at scale, the remaining vulnerabilities for the same software will—at large—be left untouched by mass attackers. Defenders can then concentrate efforts in different parts of the system (e.g., a different software component). We enucleated several empirical hypotheses of attacker behavior that are direct consequences of this model, with a direct impact on the corresponding risk management process by defenders.

Mathematically, we model the timing of effort by the attacker as a dynamic programming problem and then, for the purpose of empirical analysis, restrict it to an attacker focusing on the “next” update of their exploit portfolio (Section 3). To evaluate empirically the time delays in between these exploit updates we derive, directly from the theoretical model, a regression model of equilibrium update times (Section 8) regressing over vulnerability and attacked system characteristics. We then use results from the regression model to test several empirical hypotheses for the regression variables emerging naturally from the theoretical model (summary in Table V). To empirically validate our model, we leverage on big data analysis and the Worldwide Intelligence Network Environment (WINE) data set (Dumitras & Shou, 2011) spanning two million attack signatures recorded in the wild by Symantec, a large security firm (Section 6). In the empirical study, we control for several factors related to the characteristics of the user and their system (e.g., user geographical locations). We discuss the results of the empirical analysis (Section 7) and conclude the article by outlining implications for theory and practice (Section 10).

**Table I:** Parameters and Variables from the Model

Parameter	Description
Variable	
t	Continuous time index.
$T_{i}$	An update time when an attacker updates the vulnerabilities, indexed by i∈{0,1,2,…}. n is the last update (if it is finite).
V	The universe of known vulnerabilities affecting all systems.
N	The total number of target machines affected by vulnerabilities in V.
V	The subset of vulnerabilities in V identified by attackers for exploitation.
$\theta_{V}$	The fraction of N affected by V∈V.
$r(t,N_{V},V)$	Revenue function from successful attacks.
c(t,V)	Variable cost function for deploying attacks.
$C_{i}$	Fixed cost of adding at $T_{i}$ new vulnerabilities to be exploited by the attacker.
λ	Arrival rate of vulnerability patches to the universe of systems.
δ	Discount rate of the attacker.
$\Pi_{V}\left(t\right)$	Profit function for a given set of vulnerabilities V.

**Table II:** Variables Included in Our Data Set

Variable	Description
${\mathrm{CVE}}_{1,2}$	The identifier of the previous and the current vulnerability v exploited on the user's machine.
T	The delay expressed in fraction of year between the first and the second attacks.
N	The number of detected attacks for the pair *previous attack, actual attack*.
U	The number of systems attacked by the pair.
Compl	The Complexity of the vulnerability as indicated by its CVSS assessment. Can be either High, Medium,or Low as defined by CVSS(v2) Mell, Scarfone, and Romanosky (2007).
Imp	The impact of the vulnerability measured over the loss in confidentiality, integrity, and availability of the affected information. It is computed on a scale from 0 to 10 where 10 represents maximum loss in all metrics, and 0 represents no loss. Mell et al. (2007).
Day	The date of the vulnerability publication on the National Vulnerability Database.
Sw	The name of the software affected by the vulnerability.
Ver	The last version of the affected software where the vulnerability is present.
Geo	The country where the user system is at the time of the second attack.
Hst	The profile of the user or “host.”
Frq	The average number of attacks received by a user per day.
Pk	The maximum number of attacks received by a user per day.

**Table III:** Summary Excerpt from Our Data Set

${\mathrm{CVE}}_{1}$	${\mathrm{CVE}}_{2}$	T	U	N	Geo	Hst
2003‐0533	2008‐4250	83	186	830	IT	Up
2003‐0818	2003‐0818	146	1	1	US	Rm
2003‐0818	2009‐4324	616	1	1	CH	Ev
2003‐0818	2009‐4324	70	52	55	US	Ev

*Note*: We provide an example useful to interpret these data. Looking at the third row, one WINE system (U= 1) located in Switzerland (Geo= CH) suffered only once (N= 1) from an attack targeting the vulnerability ${\mathrm{CVE}}_{2}=$ CVE‐2009‐4324 that was preceded by an attack targeting ${\mathrm{CVE}}_{1}=$ CVE‐2003‐0818 almost two years earlier (T= 616). In the fourth row, U= 52 systems in the United States (Geo= US) received N= 55 times the first attack on ${\mathrm{CVE}}_{1}$ followed by the second attack on ${\mathrm{CVE}}_{2}$ just two months apart (T= 70). In both cases, the systems considered are of type EVOLVE, indicating that the affected systems have been upgraded and moved from some other country to the country listed in Geo during our observation period.

**Table IV:** Sample Attack Scenarios and Compatibility with Work‐Aversion Hypothesis

Type	Condition	Hypothesis
$A_{1}$: First attack and second attack affect precisely the same vulnerability and, consequently, the same software version	${\mathrm{CVE}}_{1}={\mathrm{CVE}}_{2}$	Often for Hypothesis 3 as $T^{★}\to \infty$
$A_{2}$: First attack and second attack affect the same software but different CVEs and different software versions.	${\mathrm{CVE}}_{1}\neq {\mathrm{CVE}}_{2}$, ${\mathrm{Sw}}_{{\mathrm{CVE}}_{1}}={\mathrm{Sw}}_{{\mathrm{CVE}}_{2}}$, ${\mathrm{Ver}}_{{\mathrm{CVE}}_{1}}\neq {\mathrm{Ver}}_{{\mathrm{CVE}}_{2}}$	Less frequent for Hypothesis 1 and Hypothesis 2 as $0<T^{★}<\infty$
$A_{3}$: First and second attacks affect the same software and the same version but exploit different vulnerabilities	${\mathrm{CVE}}_{1}\neq {\mathrm{CVE}}_{2}$, ${\mathrm{Sw}}_{{\mathrm{CVE}}_{1}}={\mathrm{Sw}}_{{\mathrm{CVE}}_{2}}$, ${\mathrm{Ver}}_{{\mathrm{CVE}}_{1}}={\mathrm{Ver}}_{{\mathrm{CVE}}_{2}}$	Almost never for Hypothesis 1 as $\theta_{V\cup \{v\}}=\theta_{V}$

*Note*: We expect the majority of attacks generated by the work‐averse attacker to be of type $A_{1}$. Attack $A_{2}$ should be less frequent than $A_{1}$, as it requires to engineer a new exploit. $A_{3}$ contradicts the work aversion hypothesis and should be the least common type.

**Table V:** Summary of Predictions Derived from the Model

Model Variable	Regressor	Expectation	Hypothesis	Rationale
T	T	$\beta_{1}<0$	3 and 4	Shorter exploitation times are associated with more vulnerable systems, hence T↑⇒U↓.
C(V|v)	${\mathrm{Compl}}_{CVE2,L}$	$\beta_{2}<0$	1, 4, and 2	The introduction of a new reliable, low‐complexity exploit minimizes implementation costs, thus C↓⇒U↓.
$\theta_{V\cup \{v\}}$	${\mathrm{Imp}}_{CVE2,H}$	$\beta_{3}>0$	5 and 4	High impact vulnerabilities allow the attacker for a complete control of the attacked systems, hence $\theta_{V\cup \{v\}}↑\Rightarrow U↑$.
$r,(\theta_{V\cup \{v\}}-\theta_{V})$	${\mathrm{Imp}}_{CVE2}>{\mathrm{Imp}}_{CVE1}$	$\beta_{4}<0$	5	Selecting a higher impact exploit for a new vulnerability increases the expected revenue and increases the fraction of newly controlled systems with respect to the old vulnerability. r↑⇒U↓ and $(\theta_{V\cup \{v\}}-\theta_{V})↑\Rightarrow U↓$.

## 2 BACKGROUND

The risk analysis literature has considered the need for data‐driven cyber‐risk models numerous times (Allodi & Massacci, 2017; Rao et al., 2016). A significant obstacle of cyber‐risk models is the lack of an empirically tested *attack production model* that describes the attacker decision process (Cox Jr, 2008). A major difficulty is that attackers are very diverse and do not have any sort of centralized decision‐making process. When characterizing this process, a crucial differentiation must however be made between “Mass‐Attackers” (who focus on high‐volume of possibly low‐value targets) and “Advanced Persistent Threats” (or APTs for short) generated by highly specialized groups that target specifically few high‐value targets. Targeted cyberattacks are characterized by a strong “adversarial” connotation (Rios Insua, Rios, & Banks, 2009) where the attacker can (and has the resources to) perform sophisticated reconnaissance of the target(s), identify suitable *0‐day* attacks, and tailor the whole attack process against the specific target (Paté‐Cornell, 2012). Yet, these attackers only make up for a small fraction of the attack space (Bilge & Dumitras, 2012; Research, 2018).

By contrast, mass‐scale attacks focus on (known) vulnerabilities that remain long unpatched (Nappa, Johnson, Bilge, Caballero, & Dumitras, 2015; Research, 2018) and are concentrated among a few only of the several thousand vulnerabilities available to attackers (Allodi & Massacci, 2014; Research, 2018). Unfortunately, timely patching of all vulnerabilities is infeasible (Kotzias, Bilge, Vervier, & Caballero, 2019) due to the high costs associated with the patching decision (Verizon, 2011). This effectively makes the problem of identifying which vulnerabilities are (or are going to be) high risk a highly practical problem to solve.

To help defenders make more informed decisions, we need a *characteristic model* of the mass attackers that does not depend on idiosyncratic characteristics of the attacker (e.g., their origin or motive). Since exploit engineering is an expertise‐intensive, time‐consuming process, and the mass attackers do not generate APTs, a realistic model of the mass attacker is incompatible with models whereby the attacker can exploit any vulnerability at will. Without reconstructing this missing block, it is impossible to build predictive models to infer which vulnerabilities must be fixed immediately, and which can wait.

Defender strategies in terms of patching times have been investigated both empirically (Kotzias et al., 2019; Okhravi & Nicol, 2008) and theoretically (Serra, Jajodia, Pugliese, Rullo, & Subrahmanian, 2015b), assuming specific threat models and attack production functions. For example, software vendors may maximize profit by exploiting attacker behavior (Kannan, Rahman, & Tawarmalani, 2016); system diversification, as opposed to patching, may yield lower costs when compared to ineffective “single‐metric” patching policies (Dey, Lahiri, & Zhang, 2015). Similarly, security best practices do not necessarily lead to more robust firm security, and the relation between security and liability may also be affected by different managerial settings (Lee, Geng, & Raghunathan, 2016).

The importance of well‐grounded observations for realistic and operational models capable of supporting strategic decision making at the level of a firm or organization is of relevance across several domains, including system resilience (Guikema, McLay, & Lambert, 2015). For example, the balance between recommendation and implementation of rules and regulations aimed at reducing the attack surface of a system is delicate, and must be modulated against the threat: overregulation risks (e.g., by alienating users that are supposed to implement those policies) opening up additional attack paths exploitable by attackers. On the other hand, underregulation is also undesirable to avoid leaving important vulnerabilities open (Gisladottir, Ganin, Keisler, Kepner, & Linkov, 2017). Game theory is a popular tool to investigate these trade‐offs, but assumptions behind those model must remain realistic to derive effective “operational” recommendations (Guikema et al., 2015). Most game‐theoretic models generally consider the attacker to be potentially capable of adopting any strategy with different degrees of probability, depending on the conditions of the game (see  Do et al., 2017, for a survey). For instance, Manshaei, Zhu, Alpcan, Bacşar, and Hubaux (2013) posit a case where attacker strategies can range from fixed attack updates to adaptive strategies based on the defender's decisions (van Dijk, Juels, Oprea, & Rivest, 2013), or on expectations of the attack's *persistence* and *stealthiness* to defender detection and remediation capabilities (Smeets, 2018). Attacker/defender equilibrium forces are further analyzed by Zhang and Zhuang (2019), who study optimal defensive strategies in the presence of adaptive attackers and multiple attack types, for which different probabilities of success and impact on the defended infrastructure lead to different defensive outcomes. Whereas Zhang and Zhuang (2019) do not focus on cyberattackers, the cyber‐threat landscape of mass attackers and APT‐level attackers poses similar challenges for the strategic allocation of defensive resources. Recent papers have also focused on systemic issues; for example, Kuper, Massacci, Shim, and Williams (2020) develop a static game in which network structure plays a role in the equilibrium actions of attackers. However, the dynamics of adjustments in attacker effort has not yet been explored.

Attacks against large pools of “similar” targets (e.g., by geographical distribution, or system configuration) adapt to the state of the *population* of potential targets (as opposed to one specific target), for which attack technologies developed “ad‐hoc” are not always viable (Ransbotham & Mitra, 2009). For an attacker sensitive to the cost of engineering a technical or social exploit, not all attack types make sensible avenues for investment (Herley, 2013). This is supported by empirical evidence showing that attack tools actively used by attackers embed only an handful of exploits (Kotov & Massacci, 2013), and that the vast majority of attacks recorded in the wild are driven by only a small fraction of known vulnerabilities (Allodi, 2015; Nayak et al., 2014). Some reward must be forthcoming, as the level of effort required to implement and deliver the attack observed in the wild is not negligible, as demonstrated by the presence of an underground market where vulnerability exploits are rented to attackers (“exploitation‐as‐a‐service”) as a form of revenue for exploit writers (Grier et al., 2012). In fact, recent studies on different samples (Allodi & Massacci, 2014) have challenged the automatic transfer of the technical assessment of the “exploitability” of a vulnerability into actual attacks against end users: There is a substantial lack of correlation between the observed attack signatures in the wild and metrics (such as the Common Vulnerability Scoring System [CVSS)aintained and employed by the NIST; First.org, 2015) providing an assessment of the vulnerability severity. The current trend in industry is to use metrics such as CVSS as proxies for risk and demanding immediate action (Beattie et al., 2002), but evidence suggests this may be neither sensible, nor effective (Allodi & Massacci, 2014).

The risk analysis literature has also identified the importance of contextual factors to the realization of adversarial risks  (Rios Insua et al., 2009; Serra, Jajodia, Pugliese, Rullo, & Subrahmanian, 2015a), particularly when aiming at building probabilistic and quantitative models of attack arrival (Allodi & Massacci, 2017; Brown & Cox Jr, 2011). The current lack of a sound model describing the generation of new cyberattacks is effectively preventing the literature to fully move from a qualitative/semiquantitative risk framework to a quantitative one (Cherdantseva et al., 2016).

## 3 THE WORK‐AVERSE ATTACKER

Our model captures the update process of attacks deployed at scale by a large collection of uncoordinated mass attackers. Importantly, mass attackers seldom build their own independent attack technologies, but rather fetch attacks from a shared pool of available attacks (e.g., by acquiring them through the underground economy; Allodi, 2017; Grier et al., 2012; Kotov & Massacci, 2013). The intuition behind our model is that mass attackers with similar objectives (e.g., to install botnet malware on Windows systems in the United States) will have, at large, to move over to the next available attack when those targets, on average, cannot be infected anymore with the old attack technology. Hence, the process with which attackers of the same “type” update their attack portfolio can be captured by a unified model considering all attackers of that type jointly. We model attackers to be risk neutral, and to gain revenue by making both fixed and variable cost investments in attacking a large group of independent target systems. The parameters determining the optimization problem faced by the attacker are presumed to be independent and identically distributed across the collection of attackers.

Each attacker starts their activities at time t=0 by identifying a subset of vulnerabilities V⊂V from a universe V affecting a number of target systems N. A fraction $\theta_{V}$ of the N systems is affected by V and would be compromised by an exploit in absence of security countermeasures. Targets deploy patches, update systems, or use new signatures in antivirus or IPSs whose relative effect on attack success has been discussed in extant literature (Chen, Kataria, & Krishnan, 2011; Nappa et al., 2015). Such arrival rate is typically uncorrelated with vulnerability discovery as it depends on external scheduling by software vendors, or testing in companies. For example, a recent empirical study (Kotzias et al., 2019) showed that it may take more than six months to arrive at patching 90% of the vulnerable systems. Denoting the mitigation and patching adoption rate by λ, we define the number of systems impacted by vulnerabilities in V at time t as

(1) $$N_{V}\left(t\right)=N\theta_{V}e^{-\lambda t}.$$

To engineer the exploits for the vulnerabilities V, the attacker will pay an upfront cost C(V|∅) and has an instantaneous stochastic profit function of

(2) $$\Pi_{V}\left(t\right)=\left[r\left(t,N_{V}\left(t\right),V\right)-c\left(t,V\right)\right]e^{-\delta t}.$$

The function $r(t,N_{V},V)$ is a stochastic revenue component that accounts for the probability of establishing contact with a vulnerable system (Franklin, Paxson, Perrig, & Savage, 2007), making a successful infection given a contact (Allodi, Kotov, & Massacci, 2013), and monetizing the infected system (Grier et al., 2012; Rao & Reiley, 2012); the factor c(t,V) is the variable costs of maintaining the attack (payload obfuscation to avoid detection; Kotov & Massacci, 2013) or renew the domain names to prevent domain blacklisting (Stone‐Gross et al., 2009), both subject to a discount rate δ. We do not make any assumption on the accounting unit for revenues from successful attacks. For instance, revenues can also be in the form of kudos on hacker forums (Ooi, Kim, Wang, & Hui, 2012), or revenues from trading victim's assets in black markets (Campobasso & Allodi, 2020).

At some point, the attacker might decide to perform a refresh of the attacking capabilities by introducing a new vulnerability and engineering its exploit by incurring an upfront cost of C(v|V). This additional vulnerability will produce a possibly larger revenue $r(t,N_{V\cup \{v\}}\left(t\right),V\cup \left\{v\right\})$ at a marginal cost c(t,V∪{v}). As the cost of engineering an exploit is large with respect to maintenance (C(v|V)≫c(t,V∪{v})) and neither successful infection (Allodi et al., 2013) nor revenues are guaranteed (Allodi, Corradin, & Massacci, 2015; Rao & Reiley, 2012), the attacker faces a problem of deciding action versus inaction in the presence of fixed initial costs as described by Stokey (2008). The optimal strategy is to deploy the new exploit only when the old vulnerabilities no longer guarantee a suitable expected profit. This decision problem is then repeated over time for n newly discovered vulnerabilities, and n refresh times denoted by $T_{i}$. Model parameters are summarized in Table I.

We denote by $C_{0}=C\left(V|\emptyset \right)$ the initial development cost and by $C_{i+1}\equiv C\left(v_{i+1}|V\cup \left\{v_{1}\text{…}v_{i}\right\}\right)$ the cost of developing the new exploits, given the initial set V and the additional vulnerabilities $v_{1}\text{…}v_{i}$. We denote by $N_{i}\left(t\right)\equiv N_{V\cup \{v_{1},\text{…},v_{i}\}}\left(t\right)$ the number of systems affected by adding the new vulnerability at time t. We make no assumption on the particular order over the vulnerabilities $v_{i}$. We simply assume that there is some sequence in which they are engineered and that sequence will be determined empirically. Similarly, we define $r_{i}\left(t\right)$ and $c_{i}\left(t\right)$ as, respectively, the revenue and the marginal cost of the vulnerability set $V\cup \{v_{1},\text{…},v_{i}\}$. The critical tipping point is when the instant marginal cost is equal to the instant marginal revenue $r_{i}\left(T_{i+1},N_{i}\left(T_{i+1}\right)\right)=c_{i}\left(T_{i+1}\right)$, and at this point, the attacker will need to refresh the set of exploited vulnerabilities in order to continue making a profit, thus identifying all action points $T_{i+1}>T_{i}$. Since the maintenance of malware, for example, through “packing” and obfuscation (i.e., techniques that change the aspect of malware in memory to avoid detection) is minimal does not depend on the particular vulnerability (Brand, Valli, & Woodward, 2010; Kotov & Massacci, 2013), and can be automated in matter of minutes (Castro, Schmitt, & Rodosek, 2019), the maintenance costs are negligible relative to the fixed costs of updating, hence $c_{i}\left(t\right)\ll C_{i}$, and thus the next interval tends to infinity, $T_{n+1}\to \infty$. A change of technological constraints (e.g., widely deployed detection techniques capable of identifying any variant of the same attack) would require to at least partially revise these assumptions, and therefore the model, in the future.

Empirical evidence indicates the mass attacker faces a decision problem with repeated peak actions with random revenues followed by long periods of quasi‐inaction (Allodi, 2015; Nayak et al., 2014). Whereas problems of this type are oftentimes analytically intractable, Stokey (2008) provides a framework offering a series of approximating solutions that can be applied to generic formulations of processes with “quasi‐inaction.”^1^ Accordingly, we assume an history‐less payoff with a risk‐neutral preference so that expected pay‐off and expected utility coincide and risk preferences are then encapsulated in the discount factor. See Stokey (2008) and Birge and Louveaux (2011) for a discussion.

The expected payoff from deployed malware at time t (where t≥T is the amount of time since the attacker updated the menu of attacks by engineering new exploits at time T) is then as follows:

(3) $$\begin{array}{c} r\left(t,N_{V\cup \{v\}}\left(t\right)\right)=rN\left(\theta_{V}e^{-\lambda t}+\left(\theta_{V\cup \{v\}}-\theta_{V}\right)e^{-\lambda (t-T)}\right). \end{array}$$

The first term in the parentheses measures the systems' vulnerability to the set V of exploited vulnerabilities that have been already partly patched, while the second term accounts for the new, alternative systems that can now be exploited by adding v to the pool of vulnerabilities being targeted. For the latter systems, the unpatched fraction restarts from one at time T. Together the terms deliver the instant expected revenue process across their campaign. The attackers' decision problem is then to establish the timing of when to implement v. Indeed, from an empirical observation of malware in the wild, it is clear that technology diffusion is not strictly continuous (Allodi & Massacci, 2013; Bilge & Dumitras, 2012; Nayak et al., 2014).

The attacker faces the problem of choosing a sequence of update times indexed from n→∞

(4) $$\begin{array}{ccc} \left\{T_{1}^{*},\text{…},T_{n}^{*}\right\} & = & \underset{\left\{T_{1},\text{…},T_{n}\right\}}{\arg\max}\sum_{i=0}^{n}\left(\Pi \left(T_{i+1},T_{i}\right)-C_{i}\right)e^{-\delta T_{i}}, \end{array}$$

(5) $$\begin{array}{ccc} \Pi (T_{i+i},T_{i}) & & =\int_{T_{i}}^{T_{i+1}}\left(r_{i}\left(t,N_{i}\left(t\right)\right)-c_{i}\left(t\right)\right)e^{-\delta t}dt\\ & & =\frac{rN}{\lambda +\delta }\cdot \left(\theta_{i}-\theta_{i-1}+\theta_{i-1}e^{-\lambda T_{i}}\right)\cdot \\ & & \left(1-e^{-\left(\lambda +\delta \right)\left(T_{i+1}-T_{i}\right)}\right), \end{array}$$

where $\theta_{-1}\equiv 0$, $\theta_{0}\equiv \theta_{V}$, and $\theta_{i}\equiv \theta_{V\cup \{v_{1}\text{…}v_{i}\}}$.

Note that, from the above formulation, relatively large discount rates lead to an exponential decrease of the impact of update decisions. This is a common observation also in dynamic planning problems (see  DeGroot, 2005, for an extended discussion), and provides us with a clear rationale for restricting our attention to cases when $T_{1}^{*}>0$ (since the optimal subsequent update $T_{2}^{*}$ is then sufficiently far into the future to not disturb the first update $T_{1}^{*}$). Indeed, for cases when $T_{1}^{*}>0$ and $T_{2}^{*}\to \infty$, a closed‐form solution for the next update is easily obtained by manipulation of the first‐order conditions for $T_{1}^{*}$ holding $T_{2}^{*}$ as constant. Due to the high uncertainty of future vulnerability discoveries and achievable attack reliability (Allodi & Massacci, 2014; Bozorgi, Saul, Savage, & Voelker, 2010), it is reasonable to assume that attackers generally operate under an assumption of sufficiently high discount rates for the above to hold.

Proposition 1 A risk‐neutral attacker focusing on the next update with decreasing effectiveness due to patching and antivirus updates, a negligible cost of maintenance for each exploit, and a marginal profit at least equal to the marginal revenue for each machine ($\partial \Pi /\partial T\geq r(0,N_{V}\left(0\right),V)/N_{V}\left(0\right)$) will renew her exploit at time $T^{★}$

(6) $$\begin{array}{c} T^{★}=\frac{1}{\delta }\log\left(\frac{C(v|V)}{r}-\frac{\delta }{\lambda +\delta }\left(\theta_{V\cup \{v\}}-\theta_{V}\right)N\right) \end{array}$$

under the condition $\frac{C(v|V)}{rN}\geq \frac{1}{N}+\frac{\delta }{\lambda +\delta }\left(\theta_{V\cup \{v\}}-\theta_{V}\right)$.

This condition provides a lower bound for the trade‐off provided by the cost of introducing a new exploit (C(v|V)), and the expected revenue across infected systems (rN); as the latter decreases as systems get patched, the cost of introducing a new exploit becomes justified and leads to the satisfaction of the condition, and hence to the existence of an optimal update time $T^{*}$. The proof for Equation (6) is available in the Supporting Information (Allodi, Massacci, & Williams, 2017).

The *“all–powerful” attacker* is still admitted as a particular case when the attacker cost function $\frac{C(v|V)}{r}$ for weaponizing a new vulnerability goes to zero. In this case, Proposition 1 predicts that the attacker could essentially deploy the new exploit at an arbitrary time [0,+∞] even if the new exploit would not yield a large impact.

## 4 EMPIRICAL MODEL DERIVATION

**Fig 1:**
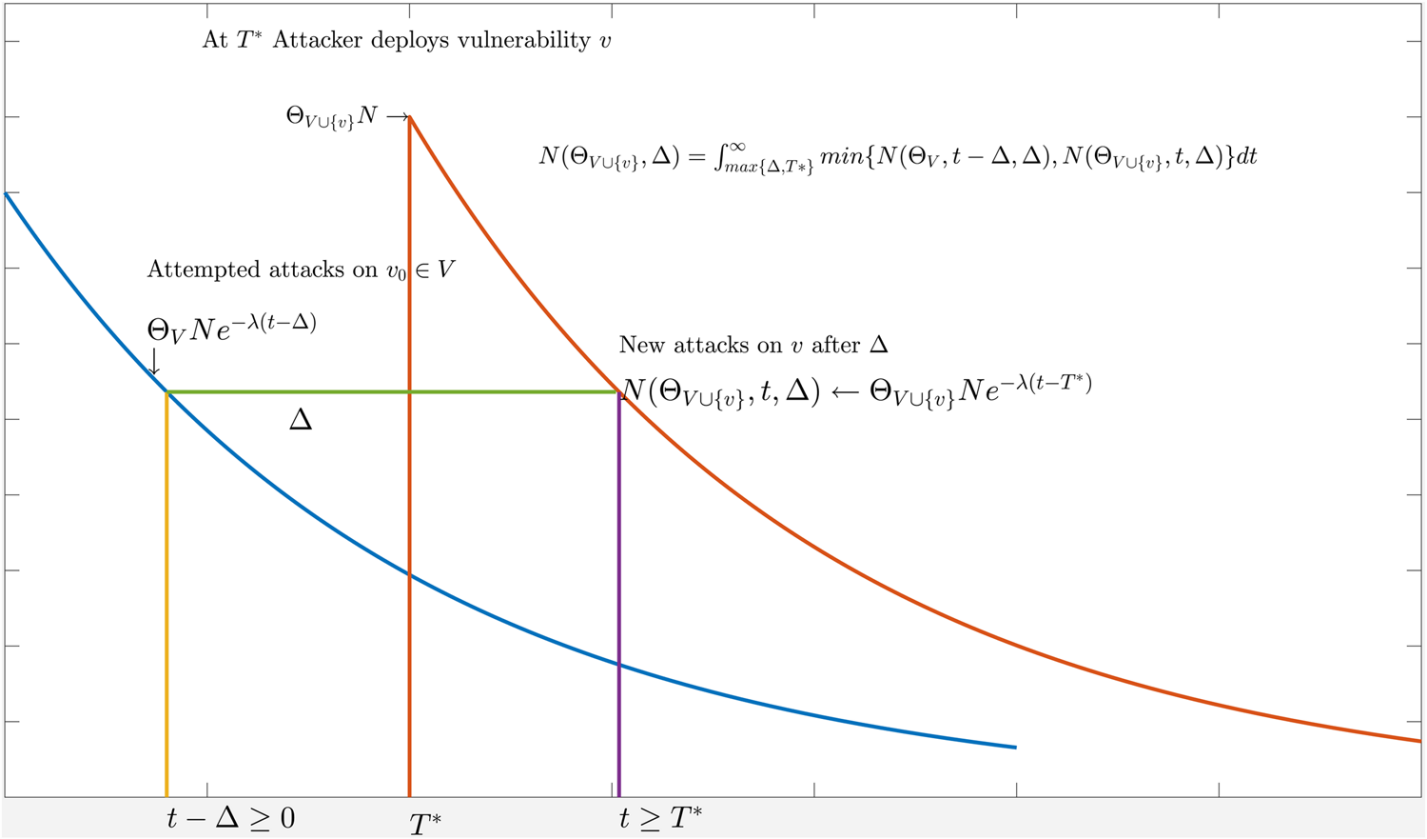
Computing the delay (T) between attacks against different vulnerabilities. *Note*: Change in the number of attacked systems for two attacks against different systems Δ=T days apart. The first attack happens at t−T≥0 and the number of attacked systems $U(\Theta_{V}\cup \left\{v\right\},t,T)$ is derived from Equation (1) as $\Theta_{V}Ne^{-\lambda (t-T)}$. The number of systems attacked by the new exploit introduced at $T^{★}$ is derived as $U\left(\Theta_{V\cup \{v\}},t,T^{★}\right)=N\Theta_{V\cup \{v\}}e^{-\lambda (t-T^{★})}dt$.

To empirically evaluate this model, we would need to measure the time $T^{★}$ of introduction of new exploits by attackers at scale. This is clearly not possible without the attackers' cooperation. To avoid this identification problem, we use the time in between two consequent attacks T as a suitable proxy. Fig. 1 reports a pictorial representation of the transformation. Each curve represents the decay in time of number of attacks against two different vulnerabilities. The first attack (blue line) is introduced at t=0, and the second (red line) at $t=T^{★}$. The number of received attacks is described by the area below the curve. Let $U(\Theta_{V}\cup \left\{v\right\},t,T)$ represent the number of systems that receive two attacks T days apart, at times t−T and t, respectively. Setting the number of attacks at time t−T as $U\left(\theta_{v},t-T\right)=N\theta_{v}e^{-\lambda (t-T)}$ and the attacks received on the second vulnerability at time t as $U\left(\theta_{V\cup \{v\}},t\right)=N\theta_{V\cup \{v\}}e^{-\lambda (t-T^{★})}$, we obtain

(7) $$\begin{array}{ccc} U(\theta_{V\cup \{v\}},t,T) & = & \min\left(N\theta_{v}e^{\lambda T},N\theta_{V\cup \{v\}}e^{\lambda T^{★}}\right)\cdot \\ & & \cdot \int_{\max(T,T^{★})}^{\infty }e^{-\lambda t}dt. \end{array}$$

Solving for the two cases $T^{★}>T$ and $T^{★}<T$, we formulate the following claim:

Claim 1 The sign of the coefficient for T oscillates from positive to negative as T increases.

(8) $$\begin{array}{ccc} \log U & & \left(\theta_{V\cup \{v\}},t,T\right)=\log\frac{N}{\lambda }\\ & & +\left\{\begin{array}{cc} -\lambda \left(T^{★}-T\right)+\log\theta_{v} & \text{if}\;T^{★}>T\\ +\lambda \left(T^{★}-T\right)+\log\theta_{V\cup \{v\}} & \text{if}\;T^{★}<T \end{array}\right. \end{array}$$

**Fig 2:**
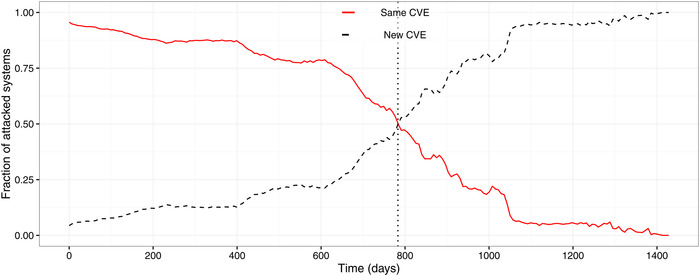
Distribution of time between of subsequent attacks with similar signatures. *Note*: Fraction of systems receiving the same attack repeatedly in time (red, solid) compared to those receiving a second attack against a different vulnerability (black, dashed). The vertical line indicates number of days after the first attacks where it is more likely to receive an attack against a new vulnerability rather than against an old one

The proof is available in the online SSRN report (Allodi et al., 2017). As the empirical evidence indicates (see Fig. 2) that T is substantial, we infer $T^{★}<T$. Hence, by substituting the corresponding term for $T^{★}$ from Equation (6), we obtain the number of expected attacked systems after T days:

(9) $$\begin{array}{ccc} \log U & & =-\lambda T+\log\frac{N}{\lambda }+\log\theta_{V\cup \{v\}}\\ & & +\lambda \left[\frac{1}{\delta }\log\left(\frac{C(v|V)}{r}-\frac{\delta }{\lambda +\delta }\left(\theta_{V\cup \{v\}}-\theta_{V}\right)N\right)\right]. \end{array}$$

## 5 HYPOTHESIS DERIVATION

Proposition 1 and Equation (9) can be used to define suitable empirical hypotheses. At first, we notice that for two vulnerabilities of the same software version, $\theta_{V\cup \{v\}}=\theta_{V}$, and therefore we hypothesize the following,

Hypothesis 1 A work‐averse attacker has only one reliable exploit per software version.

The practical implications for mitigation mechanisms is significant: If attackers are likely to exploit different vulnerabilities of the same software, the *only* secure solution would be update the whole system. If only one vulnerability is exploited, one can resort to filtering those specific attacks by an intrusion prevention system (IPS), or deploying other vulnerability‐specific defenses at the system level. For industrial control systems, that cannot be updated, deploying an IPS is the approach used in practice. Hypothesis 1 shows that this may actually be an effective defensive strategy, vastly reducing the scope of the threat caused by mass attackers. In turn, this simplifies decisions on defensive resource allocation and allows defenders to more effectively focus on defenses for different attackers and attack types (e.g., APT protection), as opposed to wasting resources to protect against *all* vulnerabilities for which an attack at scale will not, most likely, materialize (Zhang & Zhuang, 2019).

When two vulnerabilities cover essentially the same fraction of the population ($\theta_{V\cup \{v\}}-\theta_{V}\approx \varepsilon$), a low cost would make quick exploit development more appealing for an attacker because it would match the marginal condition ($C(v|V)/rN\leq \delta /\left(\lambda +\delta \right)(\theta_{V\cup \{v\}}-\theta_{V})\approx \varepsilon$) when the attacker would consider deploying an exploit to have a positive marginal benefit. To capture this aspect, we observe that we have a suitable proxy among our parameters to capture development costs. The technical term of “exploit complexity” used by the CVSS standard refers to the technical possibility of easily developing a reliable exploit that works at all times without a “complex” engineering effort to cater for random factors (e.g., the specific system configuration, memory layout) that are outside the control of the attacker. Hence low complexity significantly decreases the fixed costs of development.

Hypothesis 2 A work‐averse attacker has exploits with similar low complexity for similar popular software.

Assuming costs and rewards over $\left[0,T_{i}^{*}\right]$ are measured in the same numèraire and approximately within the same order of magnitude, the model implies that the discount factor (the term 1/δ in Equation (6)) plays a leading role in determining the optimal time for the new exploit deployment. Microeconomics literature (Frederick, Loewenstein, & O'donoghue, 2002) sets $e^{\delta }-1$ to vary between 1% and 20%. Hence, a lower bound on $T_{1}^{*}$ would be ≈[100,400] when time is measured in days.

Hypothesis 3 The time interval after which a new exploit would economically dominate an existing exploit is large (e.g., $T_{1}^{*}>100$ days).

Since $\partial T^{★}/\partial \left(\left(\theta_{v}-\theta_{V}\right)N\right)<0$, a larger number of attacked systems U on *different* versions ($\theta_{v}\neq \theta_{V}$) would imply a lower delay T (as there is an attractive number of new systems that guarantee the profitability of new attacks). In contrast, the baseline rate of attacks impacts negatively the optimal time T as $\partial T^{★}/\partial \left(\theta_{V}N\right)>0$, since a larger pool of vulnerable machines makes it more profitable to continue with existing attacks (as per Hypothesis 1). The unconditional fraction of attacked systems with new updates from the WINE data set is illustrated in Fig. 2, where the crossover point of half the systems receiving attacks with the same signature but a new vulnerability targeted is around 800 days. It shows the key idea behind Hypothesis 4: If a good old exploit works, attackers will keep using it for a long time, even if a new exploit could be used.

Hypothesis 4 The possibility of launching a large number of attacks against systems for which an exploit already exists lengthens the time for weaponizing a new vulnerability ($N\cdot ({\mathrm{Ver}}_{0}={\mathrm{Ver}}_{v})↑$ implies T↑), whereas an increase in potential attacks on different systems is an incentive toward a shorter weaponization cycle ($N\cdot ({\mathrm{Ver}}_{0}\neq {\mathrm{Ver}}_{v})↑$ then T↓).

When considering the effects of costs, we observe that, as $\partial T^{★}/\partial C\left(v|V\right)>0$, the presence of a vulnerability with a low attack complexity implies dC(v|V)<0, and therefore reflects a drop in the delay T between the two attacks. We have already discussed this possibility as Hypothesis 2. As for revenues, $\partial T^{★}/\partial r<0$ implies that a lower profit results in a longer time before it makes sense to engineer a new exploit targeting a new vulnerability. When the time to engineer a new exploit is substituted into the equation of the number of attacked machines that are needed to make a profit, a dual phenomenon takes place: An increase in revenue per attack means that less machines are needed to achieve the profit condition. We however cannot precisely measure the increase in revenues, as of course no telemetry data can reveal the exact revenue extracted from a system.^2^

Hypothesis 5 Vulnerabilities with higher impact increase revenue and therefore decrease the number of attacks (${\mathrm{Imp}}_{{\mathrm{CVE}}_{2}}>{\mathrm{Imp}}_{{\mathrm{CVE}}_{1}}$ implies U↓).

## 6 DATA SET

To reconstruct the delay between arrival of multiple (identical, new) attacks on real systems, we build a data set where each row is a pair of attacks (targeting the same or different vulnerabilities) registered on similar systems deployed worldwide. The objective is to construct a data set that represents the decisions of attackers of the same “type” (see discussion on the model intuition at the beginning of Section 3) to update the attacked vulnerability (e.g., because those attackers target MS Windows machines in a specific region, and old attacks became ineffective against these targets). To construct these data, we merge information from three data sources:

First, the National Vulnerability Database (NVD) is the vulnerability database maintained by the US NIST. Known and publicly disclosed vulnerabilities are published in this data set along with descriptive information such as publication date, affected software, and a technical assessment of the vulnerability as provided by the CVSS. Vulnerabilities reported in NVD are identified by a Common Vulnerabilities and Exposures identifier (CVE‐ID) that is unique for every vulnerability.

Second, the Symantec threat report database (SYM) reports the list of attack signatures detected by Symantec's products along with a description in plain English of the attack. Among other information, the description reports the CVE‐ID exploited in the attack, if any.

Third, the WINE, maintained by Symantec, reports attack signatures detected in the wild by Symantec's products. In particular, WINE is a representative, anonymized sample of the operational data Symantec collects from users that have opted in to share telemetry data (Dumitras & Shou, 2011). WINE comprises attack data from more than one million hosts, and for each of them, we are tracking up to three years of attacks. Attacks in WINE are identified by an ID that identifies the attack signature triggered by the detected event according to Symantec's threat database. To obtain the exploited vulnerability, we match the attack signature ID in WINE with the CVE‐ID reported in SYM.

The data extraction involved three phases: (1) reconstruction of WINE users' attack history, (2) building the controls for the data, and (3) merging and aggregating data from (1) and (2). Because of user privacy concerns and ethical reasons, we did not extract from the WINE data set any potentially identifying information about its hosts. For this reason, it is useful to distinguish two types of tables: tables *computed* from WINE, namely, intermediate tables with detailed information that we use to build the final data set; and *extracted* tables, containing only aggregate information on user attacks that we use in this research. The full list of variables included in our data set is described in Table II. The full data set computed from WINE was collected in July 2013 and is available for sharing at Symantec Research Labs (under NDA clauses for access to the WINE repository) under the reference *WINE‐2012‐008*. A full replication guide is also available in Allodi et al. (2017).

We are interested in the new vulnerability v whose mass exploit is being attempted in the wild after an exploit for V vulnerabilities have been already engineered and attempted in the recent past. Our goal is to empirically evaluate whether this past is indeed more or less recent. To do so, we initially (1) extract from WINE two attack signatures received by a system (host) monitored by Symantec at different moments in time, (2) associate each attack signature to the corresponding vulnerability whose exploit is attempted (Combining WINE, SYM, and NVD), and (3) collect from WINE some features of the host, which suffered such attacks as control variables. We use the host's profile in terms of countries it connects to the Internet from, whether the host moves geographically, and whether the host upgraded to a new version of the operating system because users with profiles that change in time may look different to the attacker, and may therefore be subject to different attacks and attack volumes (Chen et al., 2011; Baltazar, 2011; Kotov & Massacci, 2013).

Table III reports an excerpt from the data set, with only selected columns for brevity. Each row represents a pair of detected attack signatures. The columns ${\mathrm{CVE}}_{1}$ and ${\mathrm{CVE}}_{2}$ report, respectively, the CVE‐ID of the attacked vulnerability in v and in the novel attack against V. Column T reports the time delay, measured in days, between the two attacks. Column N reports the overall number of attacks detected for ${\mathrm{CVE}}_{2}$ after an attack against ${\mathrm{CVE}}_{1}$; U reports the number of single systems receiving the same pair of attacks. Column Geo reports the country in which the second attack was recorded. Finally, Hst reports the type of user affected by the attack. Additional information regarding both attacked CVEs is extracted from the NVD (not reported in Table III): For each CVE, we collect publication date (Day), vulnerable software (Sw), last vulnerable version (Ver), and an assessment of the Compl of the vulnerability exploitation and of its Imp, provided by CVSS (v2).

As we mentioned, we associate an attack signature to the corresponding CVE‐ID by combining information from WINE with Symantec own database of attack signatures (SYM). However, attack signatures as reported by Symantec have varying degrees of generality, meaning that they can be triggered by attacks that targets different vulnerabilities but still follow some common pattern. For this reason, some signatures reference more than one vulnerability. In this case, we have no means to know which of the vulnerabilities was effectively targeted by the attack. Of 1,573 different attack signatures, 112 involve more than one vulnerability; to avoid introducing counting errors on the number of attacks per CVE, we dropped these attack signatures from further consideration.

## 7 EMPIRICAL ANALYSIS

Prior to conducting any correlative analysis, we illustrate some scenarios that provide *prima facie* statistical evidence on the validity of the hypotheses identified from our theoretical model.

According to Equation (6), the attacker, will *postpone* the choice of weaponizing a vulnerability v if the ratio between the cost of developing the exploit and the maximal marginal expected revenue is larger than the discounted increase in the fraction of exploited vulnerabilities, namely, $C(v|V)/rN>\delta /\left(\lambda +\delta \right)(\theta_{V\cup \{v\}}-\theta_{V})$. Empirically, this means that the attacker should prefer to (i) attack the same vulnerability multiple times rather than for only a short period of time and (2) create a new exploit only when they want to attack a new software version. To evaluate these scenarios, we identify three types of attack pairs that are summarized in Table IV: In the first type of attack pair ($A_{1}$), the first attack and the second attack affect the same vulnerability and, consequently, the same software version; in the second pair ($A_{2}$), the first attack and the second attack affect the same software, but different CVEs and different software versions; finally in the third pair, the first and second attacks affect the same software and the same version but exploit different vulnerabilities ($A_{3}$). According to our hypothesis, we expect that attacks of type $A_{1}$ should be more common than $A_{2}$ (in particular when the delay between the attacks is small), while $A_{3}$ should be the least common of the three.

**Fig 3:**
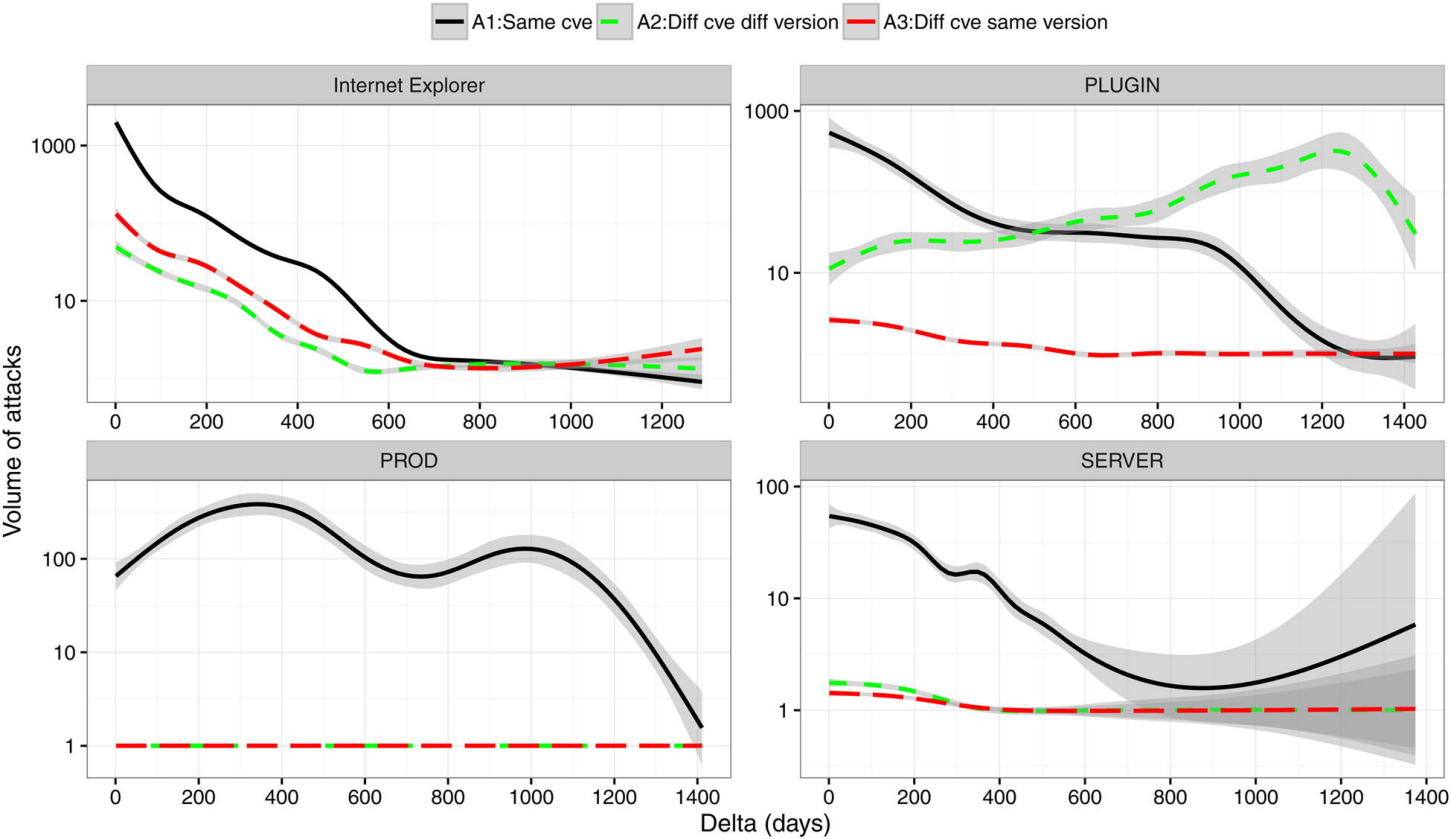
Loess regression of volume of attacks in time. *Note*: Volume of received attacks as a function of time for the three types of attack. $A_{1}$ is represented by a solid black line, $A_{2}$ by a long‐dashed red line, $A_{3}$ by a dashed green line. The gray areas represent 95% confidence intervals. For Internet Explorer vulnerabilities, the maximum T between two attacks is 1,288 days; for SERVER it is 1,374 days; PROD 1,411; PLUGIN 1,428. This can be determined by the timing of first appearance of the attack in the WINE database.

To evaluate the relative ordering of these attack types, it is important to consider that users have diverging models of software security (Wash, 2010), and different software have different update patterns, frequencies, and attack vectors (Nappa et al., 2015; Provos, Mavrommatis, Rajab, & Monrose, 2008). For example, an attack against a browser may only require the user to visit a webpage, while an attack against a word processing application may need the user to actively open a file on the system (see also the definition of the Attack Vector metric in the CVSS standard). As these clearly require a different attack process, we further classify Sw in four categories: SERVER, PLUGIN, PROD(‐ductivity), and Internet Explorer (the only browser represented in our WINE data). The categories are defined by the software names in the database. For example, SERVER environments are typically better maintained than “consumer” environments and are often protected by perimetric defenses such as firewalls or IDSs. This may in turn affect an attacker's attitude toward developing new exploits. This may require the attacker to engineer different attacks for the same software version in order to evade the additional mitigating controls in place. Hence we expect the difference between $A_{2}$ and $A_{3}$ to be narrower for the SERVER category. Fig. 3 reports a fitted curve of targeted machines as a function of time by software category. As expected, $A_{1}$ dominates in all software types. The predicted order is valid for PLUGIN and PROD. For PROD software, we find no attacks against new vulnerabilities for different software versions, therefore $A_{2}=A_{3}=0$. This may be an effect of the low update rate of this type of software and relatively short timeframe considered in our data set (three years), or of a scarce attacker interest in this software type. Results for SERVER are mixed: The difference between $A_{2}$ and $A_{3}$ is very narrow and $A_{3}$ is occasionally higher than $A_{2}$. Since oscillations occur within confidence intervals, they might be due to chance.

Internet Explorer is an interesting case in itself. Here, contrary to our prediction, $A_{3}$ is higher than $A_{2}$. By further investigating the data, we find that the reversed trend is explained by one single outlier pair: ${\mathrm{CVE}}_{1}=$ CVE‐2010‐0806 and ${\mathrm{CVE}}_{2}=$CVE‐2009‐3672. These vulnerabilities affect Internet Explorer version 7 and have been disclosed 98 days apart. More interestingly, they are very similar: They both affect a memory corruption bug in Internet Explorer 7 that allows for an heap‐spray attack resulting in arbitrary code execution. Two observations are particularly interesting:

1. Heap spray attacks are unreliable attacks that may result in a significant drop in exploitation success. This is reflected in the Access Complexity:Medium assessment assigned to both vulnerabilities by the CVSS v2 framework. In our model, this would imply a lower return $r(t,N_{V}\left(t\right),V)$ for the attacker, as the unreliable exploit may yield control of fewer machines among the vulnerable ones.
2. The exploitation code found on Exploit‐DB^3^ is essentially the same for these two vulnerabilities. The code for ${\mathrm{CVE}}_{2}$ is effectively a rearrangement of the code for ${\mathrm{CVE}}_{1}$, with different variable names. In our model, this would indicate that the cost C(v|V)≈0 to build an exploit for the second vulnerability is negligible, as most of the exploitation code can be reused from ${\mathrm{CVE}}_{1}$ (see the Appendix for details).

Hence, this vulnerability pair is only an apparent exception: The very nature of the second exploit for Internet Explorer 7 is coherent with our model and in line with Hypothesis 1 and Hypothesis 2. Removing the pair from the data confirms the order of attack scenarios identified in Table IV.

## 8 DATA ANALYSIS

Table V summarizes the predictions implied by the solution to the model given in Equation (9). T can be measured directly in our data set; the cost of development of an exploit C(v|V) can be estimated by the proxy variable ${\mathrm{Compl}}_{{\mathrm{CVE}}_{2}}$, as by definition the complexity associated with exploit development requires additional engineering efforts (and is thus related to an increase in development effort). We cannot directly measure the revenue r and the number of systems N affected by the vulnerability, but we can estimate the effect of an attack on a population of users by measuring the impact (Imp) of that vulnerability on the system: Higher impact vulnerabilities (i.e., ${\mathrm{Imp}}_{CVE2}>{\mathrm{Imp}}_{CVE1}$) allow the attacker to control a *higher fraction* of the vulnerable system, and therefore extract higher revenue r from the attack. Similarly, the introduction of an attack with a higher impact can approximate the difference in attack penetration $(\theta_{V\cup \{v\}}-\theta_{V})N$ for the new set of exploits as it allows the attacker for a higher degree of control on the affected systems. Finally, high impact vulnerabilities (${\mathrm{Imp}}_{CVE2,H}$), for example, allowing remote execution of arbitrary code on the victim system, leave the $\Theta_{V\cup \{v\}}N$ systems under complete control of the attacker; in contrast, a low impact vulnerability, for example, causing a denial of service, would allow for only a temporary effect on the machine and therefore a lower degree of control. It is important to note that other vulnerability characteristics, such as requirements on attacker positioning (e.g., local to the system, or remote) and preexistent privileges required for the attack to work may have an impact on the decisions of an attacker. On the other hand, previous research showed that, considering mass attackers, only certain types of vulnerability are effectively exploited at scale (Allodi & Massacci, 2014): Mass attackers generally attack from remote, do not have preexistent privileges on the vulnerable system, and prefer vulnerabilities for which no user interaction is required (to avoid detection, and therefore maintain exploit functionality, for longer; Allodi, 2017). Using CVSS as the framework of reference to evaluate vulnerability characteristics, most of the variability in vulnerabilities exploited at scale is captured by the relationship between attack complexity and vulnerability impact (Allodi & Massacci, 2014). Hence, these are the main factors we capture in our model. A limitation of our data set is that we cannot ascribe a specific collection of observations to a specific individual. This unobserved variable may bias our result. To attempt to correct for this statistical feature, we identify commonalities in attacks by including a number of additional components based on the type of target victim: receiving thousands of attacks a day versus an handful a year, moving in space or upgrading their software, and geographical location. Descriptive statistics of these variables are provided in the Supporting Information (Allodi et al., 2017).

We present the estimates of Equation (9) from data in Table VI, with a number of conditioning variables. These range from just a constant (Model 1, first column) to Model 3 where we include all available conditioning variables to extract systematic attack characteristics. It is important to note that for the model to be consistent with the properties of the observed empirical data the coefficient predictions from Hypotheses 1–5 summarized in Table V must be satisfied. All predictions are confirmed by the data. We utilize two estimators as we have little information on the error structure of the regression model and we are subject to certain statistical issues caused by the right truncation of the data, that is, we do not observe T asymptotically by construction. First is a simple ordinary least squares (OLS) estimator with Huber–White standard errors and second is a robust fit model that utilizes a weighted least squares (WLS) type estimator with iterative reweighting and we implement the sandwich form standard error from the WLS iterations. The weighting function for the iterative reweighting is a bisquare function, experimentation with spectral and Andrews‐type weightings suggest the regressions are insensitive to kernel and tuning function. For the robust fit, we compute a McFadden adjusted pseudo‐$R^{2}$, which sets the numerator as the log likelihood function at the estimate and the denominator as the log likelihood of just the intercept alone. Note that it is not appropriate to compare directly the pseudo‐$R^{2}$ and the $R^{2}$ from the OLS estimates, which suggests that the model captures roughly 10% of the variation in numbers of attacked machines, as opposed to explaining 35% of the model likelihood for the pseudo‐$R^{2}$.

**Table VI:** Ordinary Least Squares and Robust Regression Results

Dependent Variable: Natural Logarithm of the Number of Attacked Machines $\log(U_{i})$												
	Model 1				Model 2				Model 3			
	OLS		Robust		OLS		Robust		OLS		Robust	
	Z1:Z8		Z1:Z8		Z1:Z8		Z1:Z8		Z1:Z8		Z1:Z8	
c	0.927	0.006	0.731	0.096	1.065	0.122	0.845	0.171	0.933	−0.106	0.783	0.039
	(0.001)	(0.003)	(0.001)	(0.003)	(0.001)	(0.003)	(0.001)	(0.003)	(0.004)	(0.005)	(0.003)	(0.004)
T	0.018	−0.051	0.012	−0.044	−0.006	−0.092	−0.003	−0.071	−0.005	−0.091	−0.004	−0.071
	(0.001)	(0.001)	(0.001)	(0.001)	(0.001)	(0.001)	(0.001)	(0.001)	(0.001)	(0.001)	(0.001)	(0.001)
${\mathrm{Compl}}_{\text{CVE2}\;L}$					−0.326	−0.479	−0.228	−0.324	−0.313	−0.464	−0.22	−0.314
					(0.002)	(0.002)	(0.001)	(0.001)	(0.002)	(0.002)	(0.001)	(0.001)
${\mathrm{Imp}}_{\text{CVE2}\;H}$									0.144	0.236	0.063	0.131
									(0.003)	(0.003)	(0.003)	(0.003)
${\mathrm{Imp}}_{\text{CVE}\;2}>{\mathrm{Imp}}_{CVE1}$									−0.088	−0.209	0.012	−0.087
									(0.003)	(0.003)	(0.002)	(0.002)
Z1: Geo North. Amer.		0.604		0.37		0.679		0.422		0.671		0.419
		(0.002)		(0.001)		(0.002)		(0.001)		(0.002)		(0.001)
Z2: Geo West. Eu.		0.155		0.105		0.17		0.116		0.163		0.114
		(0.002)		(0.002)		(0.002)		(0.002)		(0.002)		(0.002)
Z3: Hst EVOLVE		0.191		0.129		0.208		0.141		0.223		0.149
		(0.002)		(0.002)		(0.002)		(0.002)		(0.002)		(0.002)
Z4: Hst UPGRADE		0.112		0.072		0.116		0.076		0.113		0.075
		(0.002)		(0.002)		(0.002)		(0.002)		(0.002)		(0.002)
Z5: Frq HIGH		0.24		0.147		0.212		0.127		0.279		0.157
		(0.003)		(0.003)		(0.003)		(0.003)		(0.003)		(0.003)
Z6: Frq MEDIUM		0.328		0.227		0.358		0.246		0.41		0.271
		(0.002)		(0.002)		(0.002)		(0.002)		(0.002)		(0.002)
Z7: Pk HIGH		0.513		0.442		0.567		0.49		0.531		0.477
		(0.004)		(0.003)		(0.004)		(0.003)		(0.004)		(0.003)
Z8: Pk MEDIUM		0.379		0.274		0.412		0.299		0.411		0.301
		(0.003)		(0.002)		(0.003)		(0.002)		(0.003)		(0.002)
$Pseudo\;R^{2}$	–	–	0.326	0.341	–	–	0.331	0.347	–	–	0.331	0.347
$R^{2}$	0.00	0.093	–	–	0.016	0.126	–	–	0.017	0.13	–	–
F	348.66	26,551.47	–	–	18,548.25	33,422.78	–	–	9,989.88	28,915.60	–	–
Obs.	2324500	2324500	2324500	2324500	2324500	2324500	2324500	2324500	2324500	2324500	2324500	2324500

*Note*: $\begin{array}{c} \;\text{Model}\;\text{1:}\;\log\left(U_{i}\right)=\beta_{0}+\beta_{1}T_{i}+\varepsilon_{i}\\ \text{Model}\;\text{2:}\;\log\left(U_{i}\right)=\beta_{0}+\beta_{1}T_{i}+\beta_{2}{\mathrm{Compl}}_{i,CVE2,L}+\varepsilon_{i}\\ \text{Model}\;\text{3:}\;\log\left(U_{i}\right)=\beta_{0}+\beta_{1}T_{i}+\beta_{2}{\mathrm{Compl}}_{i,CVE2,L}+\beta_{3}{\mathrm{Imp}}_{i,CVE2,H}+\beta_{4}{\mathrm{Imp}}_{i,CVE2}>{\mathrm{Imp}}_{i,CVE1}\varepsilon_{i} \end{array}$The three model equations reflect the definition of the expected (log) number of affected machines after an interval T. The regression model formulation is derived from prime principle from Equation (9). The expected coefficient signs are given in Table V. For each model, we run four sets of regressions. OLS and robust regressions are provided to addresses heteroscedasticity in the data. $R^{2}$ and F‐statistics are reported for the OLS estimations. Note that the pseudo‐$R^{2}$ are computed for the robust regressions, using the McFadden‐adjusted approach $R^{2}=1-\left(\log\left(LL_{full}\right)-K\right)/\log\left(LL_{int}\right)$, where $\log(LL_{full})$ is the log likelihood for the full model minus the number of slope parameters K versus the log likelihood of the intercept alone and should not be compared directly to the OLS $R^{2}$. Coefficient estimations of the two sets of regressions are consistent. All coefficient signs for the three models reflect the work‐averse attacker model predictions, with the only exception of the estimation for T with no controls for which the prediction for $\beta_{1}$ is inverted. This may indicate that user characteristics are relevant factors for the arrival time of exploits when other factors related to the system are not accounted for. The introduction of Compl in Model 2 significantly changes the estimate for $\beta_{1}$, whereas Imp in Model 3 leaves the estimates for Compl and T unchanged. High Imp vulnerabilities tend to increase volume of attacks. We report only standard errors without starring *p*‐values as all coefficients are significant due to the number of observations in the data set. All standard errors are estimated using the Huber–White approach.

The set of OLS and robust regressions returns very similar estimations. We also experimented with various regression estimators (e.g., 2SLS, 3SLS) and they produced markedly similar results to OLS, subject to the standard caveats on misidentification. The introduction of the controls only change the sign of $\beta_{1}$ from positive to negative for Model 1. This may indicate that the type of user is a significant factor in determining the number of delivered attacks, which is consistent with previous findings (Nappa et al., 2015). Interestingly, the factor that introduces the highest change in the estimated coefficient $\beta_{1}$ for T is Compl (Model 2), whereas its estimate remains essentially unchanged in Model 3. This may indicate that the cost of introduction of an exploit has a direct impact on the time of delivery of the exploit. The coefficients for all other regressors are consistent across models, and their magnitude changes only slightly with the introduction of the controls. This observation is to be expected: User characteristics should not influence the characteristics of the vulnerabilities present on the system; as such, the distribution of attacks in the wild seems to depend mostly on system characteristics rather than user type.

The signs of coefficients for the Imp variables suggest that both impact of a new vulnerability and its relation with the impact of previous vulnerabilities have an effect on the number of attacked systems. Interestingly, a *high* impact encourages the deployment of attacks and increases the number of attacked systems, whereas the introduction of a *higher* impact vulnerability requires the infection of a smaller number of systems as revenues extracted from each machine increase. Hence, when introducing a new exploit, the attacker will preferably choose one that grants a higher control over the population of users ($\theta_{V\cup \{v\}}>\theta_{V}$) and use it against a large number of system. This is consistent with recent findings suggesting that vulnerability severity alone is not a good predictor for exploitation in the wild (Allodi & Massacci, 2014; Bozorgi et al., 2010). Other factors such as software popularity may play a role (Nayak et al., 2014).

## 9 SUMMARY OF FINDINGS AND LIMITATIONS

This article implements a model of the *Work‐Averse Attacker* as a new conceptual framing to understand cyber threats. Our model presumes that an attacker is a resource‐limited actor with fixed costs that has to choose which vulnerabilities to exploit to attack the “mass of Internet systems.” Work aversion simply means that effort for the attacker is costly (in terms of cognition and opportunity costs), hence a trade‐off exists between effort exerted on new attacking technologies and the anticipated reward schedule from these technologies. As systems in the wild get patched unevenly and often slowly in time (Nappa et al., 2015), we model the production of new vulnerability exploits following Stokey's “economy of inaction,” whereby “doing nothing” up to a certain time is the best strategy. A cost constraint driving the attacker's exploit selection strategy naturally emerges from the model. In particular, we find theoretical and empirical evidence as follows:

1. First, an attacker massively deploys only one exploit per software version. The only exception we found is for Internet Explorer; the exception is characterized by a very low cost to create an additional exploit, where it is sufficient to essentially copy and paste code from the old exploit, with only few modifications, to obtain the new one. This finding is predicted by the model and supports Hypothesis 1.
2. Second, low complexity vulnerabilities for which a reliable exploit can be easily engineered lower the production costs and favor the deployment of the exploit. This finding supports Hypothesis 2.
3. Third, the attacker deploys new exploits relatively slowly over time, driven by a slowly decreasing instantaneous profit function; empirically, we find that attacks 1,000 days apart are still driven by the same exploits in about 20% of the cases, and that the effect of the passage of time in between attacks (T) on the number of affected system is indeed negative and very small. This finding supports Hypothesis 3 and Hypothesis 4.
4. Fourth, the presence of a high impact vulnerability increases the incidence of exploitation in the wild. Similarly, gaining a higher control over attacked systems heightens the attacker's revenue and decreases the number of systems that need to be infected to balance costs. This supports Hypothesis 5.

Such findings should be considered in the framework of the limitations of the data that we have collected, and the theory we have developed. The “Work‐Averse Attacker” may be only one of the possible explanations of the distribution of exploits in the wild previously noted in the literature (Allodi, 2015; Nayak et al., 2014). For example, it could be that only a handful of individuals possess the technical skills to develop (and subsequently distribute to the mass of attackers) working exploits. The strong skew in the exploit distributions could then be explained by those individuals not being work‐averse, but only in terms of available capacity for exploit production. To evaluate this possibility in the data is hard or impossible as it would require to identify (all) exploit developers and observe the exploit production process. On the other hand, the presence of competitive underground markets where multiple actors trade different, but long lived, exploits, and malware techniques does not appear compatible with the hypothesis of only a handful of productive but time‐limited exploit developers (Allodi, 2017). Alternatively, explanations for the data could be identified in the efficiency of the *supply chain* of the components needed to engineer and deploy an attack: If finding producers of the necessary attack components (or establishing business relationships with them) is hard, the inefficiency of the required supply chain could explain the observed delays in the exploit deployment process, and the scarcity of available exploits for attacks at scale. The existence of composed services for the delivery of attacks is clear evidence of the existence of this supply chain, at least for attack provision and delivery  (Campobasso & Allodi, 2020; Grier et al., 2012). These inefficiencies could push attackers to strategize on which exploits to develop, leading to similar output dynamics as those considered in this article. A rigorous evaluation of the supply chain of cyberattacks is hard to perform (Bhalerao, Aliapoulios, Shumailov, Afroz, & McCoy, 2019), but may shed additional light on the bottlenecks or hardship of development of exploits for attacks at scale. Importantly, work‐averse dynamics may still emerge, from this setting, underlying that the complexity of the problem requires a deeper empirical understanding of the ecosystem enabling attacker operations at scale.

Other limitations concern the nature of the data. Records of attacks detected over a user's machine are necessarily conditioned over the user's proneness in receiving a particular attack. For example, a user may be inclined to open executable email attachments, but not in visiting suspicious websites. Thus, there may be a disassociation between the observed attacks and those engineered by the attacker. For our empirical data set, this limitation is mitigated by *WINE* reporting attack data on a very large representative sample of Internet users (Dumitras & Shou, 2011). Albeit we do have some system‐level information (e.g., geographic location, system evolution), we do not have all possible conditioning user variables (e.g., educational level), which are very difficult or close to impossible to gauge at the scale of data needed for this type of analysis. Similarly, software versioning information is known to be unreliable at times with respect to vulnerability existence (Nguyen, Dashevskyi, & Massacci, 2015). Further, software versions cannot be easily “ordered” throughout software types, as different vendors adopt different naming schemes for software releases (for an overview, see, e.g., Christey & Martin, 2013). We cannot therefore order software versions over time easily. Another limitation of our data set is the market penetration of Symantec. In 2016 (i.e., around the time of the data collection), Symantec self‐reported that it is the largest security vendor for the last 15 years by market share in antivirus and overall software security, and hence has a broad coverage recording attacks on customers. However, third‐party verifiable measurement of these claims are difficult, hence replication studies across different security vendors would be welcome.

## 10 CONCLUSIONS AND IMPLICATIONS

This article develops the thesis that an attacker will generally “avoid to work” until the perceived utility of the deployment of a new attack becomes positive w.r.t. expectations derived from previous attacks. This economic perspective has been previously employed in game‐theoretic approaches (Manshaei et al., 2013), and it typically considers two actors (namely, the defender and the attacker) that react to each other's strategies. The realistic threat modeling is of key importance in this context, and has been identified multiple times in the system resilience (Gisladottir et al., 2017; Guikema et al., 2015) and security (Do et al., 2017; Hewett, Rudrapattana, & Kijsanayothin, 2014) literature. This article is the first to propose and validate this approach for the “mass attacker” that deploys attacks against the vast Internet population. In this respect, this contribution provides a better theoretical and empirical understanding for the behavior of “untargeted” mass attackers: *A slow periodic update of an attacker's arsenal with selected picks of low hanging fruits* seems to be the theoretical and empirically winning strategy. This finding is particularly interesting because recent, game‐theoretic work on APTs has also shown that periodic renewal strategies might also be dominant strategies for targeted attacks (van Dijk, Juels, Oprea, & Rivest, 2013). This dominance, in the Nash equilibrium sense, remains even in the case where the attacker can reliably evaluate some characteristics of the defender's setting such as system configuration changes or an average patching rates (Nappa et al., 2015).

From the perspectives of cyber‐risk assessment, this means that several alternative strategies might be equally successful than the “upgrade to the last version” (or “do nothing” if such upgrade is not possible) strategy, which currently dominates risk mitigation best practices. For example, maintaining intrusion detection systems (IDS/IPS) signatures for the low hanging fruit vulnerabilities might be a better option than updating the software, because one IDS signature eliminates the majority of risks faced be that system; a software patch may ‘overdo‐it” by fixing more vulnerabilities than necessary at a severe functional costs (Dashevskyi, Brucker, & Massacci, 2018; Huang, Borges, Bugiel, & Backes, 2019). An important assumption in this respect is the cost of exploit maintenance being negligible as some empirical research implies (Castro et al., 2019; Kotov & Massacci, 2013). If the maintaining the success of exploits would become a significant part of the cost, attackers might still be work averse but different dynamics or mechanisms may emerge. We leave this work for future research.

Another major implication for this research work is the current policy discussion on the timing of vulnerability disclosures. The United States Department of Commerce NTIA set up multistakeholders forum to discuss procedures and timings of the vulnerability disclosure process.^4^ This discussion is not currently guided by a theoretical framework for decisionmakers to estimate effect in terms of the effective increase in risk of attacks that follows the disclosure (Mitra & Ransbotham, 2015). Our findings would indicate that there is a limited risk in additional disclosures of minor vulnerabilities for the same software version (i.e., Hypothesis 1). Further, the time/space dimension may also be relevant to evaluate from a policy perspective, for example, by asynchronously releasing patches to users or by deploying different versions across systems. By diversifying software (Chen et al., 2011; Homescu, Neisius, Larsen, Brunthaler, & Franz, 2013), the defender can effectively decrease the number of systems the attacker can compromise with one exploit, effectively making the existence conditions for Equation (6) hard to satisfy. For example, a random distribution of patches would simply decrease the fraction of attackable systems regardless of the attacker's choice in which vulnerability to exploit. Moreover, diversifying defenses may be in fact less onerous than recompiling code bases (when possible) or maintaining extremely diverse operational environments. More studies are needed to evaluate cascading effects of generalized strategies against “mass attackers” on exposure to attacks of other types (e.g., perpetrated by APT‐level attackers capable of adapting to specific system conditions). In general, a more precise and data‐grounded understanding of the attacker poses a strategic advantage for the defender (Dey et al., 2015). This article is a step in this direction.

## Supporting information

Online Appendix

## Footnotes

1 In practice, these approximations allow to pass from the Riemann stochastic integral that emerges from the problem formulation, to a standard Leibniz‐style integral so that the resulting optimization problem is more tractable.

2 A possible proxy is to consider the *technical impact* of the attack as the level of system compromise that is possible to achieve by exploiting the vulnerability. Intuitively, the higher the level of access, the greater the potential revenue the attacker could extract from the system. A possible limitation is that such information might not correspond to the actual revenue for some specific attackers. For example, vulnerabilities that only compromise the availability of a system are typically scored low according to standard metrics such as the CVSS. (First.org, 2015) Yet, for an hacker offering Distributed Denial of Service (DDoS) targeted attack against online gamers, these vulnerabilities maybe the most interesting source of revenues (Hutchings & Clayton, 2016).

3 See Exploit‐DB (http://www.exploit‐db.com, last accessed January 15, 2017.), which is a public data set for vulnerability proof‐of‐concept exploits. ${\mathrm{CVE}}_{1}$ corresponds to exploit 16547 and ${\mathrm{CVE}}_{2}$ corresponds to exploit 11683.

4 The NTIA forum can be found at https://www.ntia.doc.gov/other-publication/2016/multistakeholder-process-cybersecurity-vulnerabilities, last accessed October 2020

## APPENDIX A INTERNET EXPLORER EXPLOIT CODE

Here, we report the exploit code for ${\mathrm{CVE}}_{1}=$CVE‐2010‐0806 and ${\mathrm{CVE}}_{2}=$CVE‐2009‐3672 both affecting Internet Explorer version 7. On Exploit‐DB 5 See Exploit‐DB (http://www.exploit‐db.com, last accessed April 12, 2018.)
${\mathrm{CVE}}_{1}$ corresponds to exploit 16547 and ${\mathrm{CVE}}_{2}$ corresponds to exploit 11683.

The exploit code fragments (10+ lines of code out of 260+ for ${\mathrm{CVE}}_{1}$ and 130+ for ${\mathrm{CVE}}_{2}$) below illustrates the difference between the two exploits as scripted for the Metasploit engine. The exploit code for ${\mathrm{CVE}}_{2}$ is effectively a rearrangement of the exploit code for ${\mathrm{CVE}}_{1}$, with different variable names (e.g. by replacing j_memory with var_memory, j_shellcode with var_shellcode) and repositioned at the appropriate memory addresses (0x …).

function j_function1()

2 ...

3 j_memory = new Array();

4 var j_shellcode = unescape(...);

5 var j_slackspace = 0x86000 ‐(j_shellcode.length*2);

6 while(j_nops.length< j_slackspace/2)

7 j_nops+=j_nops;

8 for(j_counter=0; j_counter<270; j_counter++)

9 j_memory[j_counter] = j_fillblock + j_fillblock + j_shellcode;

function var_body()

2 ...

3 var var_memory = new Array();

4 var var_shellcode = var_unescape(..);

5 var var_ss = 20 + var_shellcode.length;

6 while (var_spray.length < var_ss)

7 var_spray+=var_spray;

8 var_bk = var_spray.substring(..);

9 while(var_bk.length+var_ss < 0x100000)

10 var_bk = var_bk + var_bk + var_fb;

11 for (var_i=0; var_i<1285; var_i++)

12 var_memory[var_i]= var_bk + var_shellcode;
